# IFABP and TFF3 as predictors for intestinal health in the perioperative setting in children with congenital heart disease

**DOI:** 10.3389/fcvm.2025.1633172

**Published:** 2025-10-30

**Authors:** Nicolas Boerter, Noa Judith Freudenthal, Soyhan Bagci, Tobias Kratz, Johannes Breuer, Nicole Mueller

**Affiliations:** ^1^ Department of Anesthesiology and Intensive Care Medicine, University Hospital Bonn, Bonn, Germany; ^2^ Department of Pediatric Cardiology, University Hospital Bonn, Bonn, Germany; ^3^ Department of Neonatology and Pediatric Intensive Care, University Hospital Bonn, Bonn, Germany

**Keywords:** IFABP, TFF3, intestinal perfusion, congenital heart disease, enteral feeding

## Abstract

**Introduction:**

Children with congenital heart disease (CHD) undergoing cardiac surgery are at heightened risk of gastrointestinal complications due to imbalances in systemic and pulmonary blood flow, often resulting in impaired intestinal perfusion and mucosal injury. Intestinal biomarkers such as intestinal fatty acid binding protein (IFABP) and trefoil factor 3 (TFF3) indicate intestinal damage. This study investigates intestinal fatty acid binding protein (IFABP) and trefoil factor 3 (TFF3) as biomarkers for intestinal cell damage and their potential utility in predicting clinical outcomes particularly the progression of enteral feeding post-surgery.

**Methods:**

Serum and urine samples from 85 children with congenital heart defects were analyzed pre- and postoperatively at 6 time points focusing on IFABP and TFF3. IFABP and TFF3 levels were measured via ELISA and categorized into ordinal groups based on previous pediatric reference data and observed population distributions. A composite score (TI-score) combining urinary IFABP and serum TFF3 categories was created to improve predictive strength for outcome parameters. The primary outcome was time to full enteral nutrition; secondary outcomes included duration of invasive and non-invasive ventilation, vasoactive-inotropic score (VIS), and length of hospital stay.

**Results:**

Of 85 analyzed patients, 73% showed elevated preoperative serum I-FABP levels (>336 pg/ml), with postoperative peaks at ICU admission and normalization within 60 h. The TI-score, combining both biomarkers, showed the strongest correlations with clinical outcomes such as duration of invasive ventilation (IV), duration of non-invasive ventilation (NIV), ventilation duration, time to full enteral nutrition and length of hospital stay (*p* < 0.01). TI-score at 12 h post-surgery independently predicted delayed enteral feeding and outperformed single biomarkers (AUC = 0.888; cut-off 6.5, sensitivity 84.4%, specificity 85%). Children with complex CHD or extracardiac shunts had significantly higher biomarker levels and TI-scores throughout the perioperative period.

**Conclusion:**

IFABP and TFF3 are reliable biomarkers for assessing intestinal injury in children undergoing cardiac surgery. The TI-score provides enhanced prognostic value and may serve as a practical tool to guide clinical management and nutritional planning in this vulnerable population.

## 1 Introduction

Maintaining a balanced pulmonary-to-systemic blood flow is critical in children with shunt-dependent congenital heart disease (CHD) as imbalances can compromise intestinal perfusion and oxygen delivery (1). This mismatch predisposes these patients to intestinal ischemia and related complications, including necrotizing enterocolitis (NEC), which occurs with increased incidence and mortality in this population (2, 3). During cardiac surgery, altered hemodynamics can further impair gut perfusion, leading to ischemia-reperfusion injury and mucosal damage (4, 5).

Intestinal fatty acid binding protein (IFABP), a cytosolic protein expressed predominantly in enterocytes of the small intestinal villi (6), is rapidly released into circulation upon mucosal damage and cleared renally with a plasma half-life of approximately 11 min (7). Elevated IFABP levels in blood and urine have been reported in several conditions involving intestinal injury (8–10), including in children undergoing cardiac surgery (11–13). Human models showed significant correlations between the extend in histological damage and the increase in circulating IFBAP (14) and in neonates with NEC IFABP levels correlated with the length of bowel resection (15). Intestinal trefoil factor 3 (TTF3), secreted by goblet cells of the gastrointestinal tract (16), promotes mucosal repair by facilitating epithelial restitution, angiogenesis, and tight junction stabilization (17–19). Its release increases in response to epithelial injury or inflammation, and its levels show wide inter-individual variability across biological fluids (18). Despite its recognized role in mucosal protection, TFF3 has not been comprehensively evaluated in the context of pediatric cardiac surgery.

Given the challenges of enteral nutrition in CHD patients postoperatively (20, 21) particularly due to the risk of gastrointestinal complications, we evaluated the diagnostic and prognostic utility of IFABP and TFF3 in this setting. The aim of this study was to measure IFABP and TFF3 as surrogate parameter for intestinal epithelial integrity in children undergoing CHD surgery in serum and urine to explore their diagnostic and prognostic value. We specifically evaluated their association with clinical outcomes, including time to initiation and advancement of enteral nutrition, in order to investigate their potential relevance for postoperative nutritional management. Notably, the influence of anesthesia induction on intestinal cell integrity in this context has not been previously addressed. We evaluated both IFABP and TFF3, measured in two different biological mediums (serum and urine), in children undergoing CHD surgery, to explore their combined association with clinical outcomes. We specifically assessed these biomarkers before, during and after with or without cardiopulmonary bypass (CPB), to characterize their perioperative dynamics and to explore their association with relevant clinical outcomes, including the initiation and advancement of enteral nutrition.

## 2 Material and methods

From May 2015–April 2017, 85 children aged between birth and 6 years undergoing cardiac surgery for CHD were included in the study with written informed consent from their caregivers. Children who had multiple procedures were treated as separate cases if their age, weight, and hemodynamic situation changed significantly. The ethics committee of the University of Bonn approved the study (application number 375/13), which complied with the Declaration of Helsinki.

Two pediatric cardiologists classified patients according to their underlying condition into a simple—and a complex congenital heart disease group.

The **Simple congenital heart disease** group included patients with AV Canal (AVC), ventricular septal defect (VSD), atrial septal defect (ASD), sinus-venosus-defect (SVD), aortopulmonary window (APW) and partial anomalous pulmonary venous return (PAPVR).

The **Complex congenital heart disease** group included patients with critical valvular aortic stenosis, Coarctation of the aorta (CoA) with duct-dependent circulation, hypoplastic left heart syndrome (HLHS), Hypoplastic right heart syndrome (HRHS), pulmonary atresia (PA), tricuspid atresia (TA), Ebstein anomaly, D-transposition of the great arteries (D-TGA), double outlet right ventricle (DORV), tetralogy of Fallot (TOF), and total anomalous pulmonary venous return (TAPVR).

Patients were classified as having a “Shunt” in any case of duct or BT-Shunt dependent body-/lung perfusion before surgery.

**Surgeries**: ASD repair, VSD repair, AVC repair, SVD repair, APW repair, TOF repair, Coarctation repair, arterial switch, PA-banding, BT-shunt, Norwood procedure, Glenn procedure, Fontan procedure, surgical correction of Common trunk (Rastelli procedure with PA-conduit), aortic valve repair, hypoplastic arch repair.

### 2.1 Blood and urine sampling and processing

Blood and urine specimens were collected during routine perioperative sampling at six different time points. Missing values were treated as randomly missing and were excluded pairwise from statistical analysis.

Time points for data collection included T0: on the day before the surgery; T1: before surgery, right after the induction of anaesthesia and placement of the central venous catheter and Foley catheter; T2: postoperatively after administration to the pediatric cardiac intensive care unit (PCICU); T3: 12 h, T4: 48 h and T5: 60 h after surgery.

Blood samples were taken during routine perioperative blood draws from a central venous line, an arterial line, or a peripheral venipuncture. The serum was immediately separated by centrifugation and stored in aliquots at −80 °C until analysis. Urine samples were collected via a urine collection bag or an indwelling catheter when present. Urine samples were also stored in aliquots at −80 °C until analysis. All samples were processed and stored at the Liquid Biopsy Biobank, Institute of Clinical Chemistry and Clinical Pharmacology of the University Hospital Bonn. All samples were analyzed for biomarker concentrations within 2 weeks after sampling. In case of a necessary remeasurement a fresh aliquot was used that had not been thawed before.

### 2.2 Analysis of biomarker concentrations

#### 2.2.1 IFABP

Serum and urinary IFABP concentrations were measured by ELISA according to the manufacturer's protocol (Standard range: 31,3 pg/ml to 2,000 pg/ml) (R&D Systems Minneapolis, USA).

All urine and serum samples for measurement of IFABP concentrations were diluted 1:4.

Samples above the detection limit were diluted at 1:10 and 1:100, respectively.

#### 2.2.2 TFF3

Serum and urinary TFF3 concentrations were measured by ELISA according to the manufacturer's protocol (Standard range: 7,8 pg/ml to 500 pg/ml) (R&D Systems Minneapolis, USA).

Samples for TFF3 were diluted either 1:1,000 (urine) or 1:100 (serum).

All samples were measured in duplicate for quality management.

### 2.3 Data collection

Data collection from medical records included data for enteral nutrition and hemodynamic status, ventilation and length of hospital stay (LOS).

### 2.4 Vasoactive-inotropic-score (VIS)

To measure the inotropic support, vasoactive-inotropic-score (VIS), according to Gaies et al. (22) was calculated. The calculation was adapted to the used Catecholamines.

VIS = [dobutamine (µg/kg/min)]*1+ [corotrope (µg/kg/min)]*10+ [noradrenaline (µg/kg/min)]*100+ [suprarenin (µg/kg/min)]*100+ [vasopressin (mU/kg/min]*10,000.

### 2.5 Classification into groups

Due to a broad distribution of IFABP values, samples were diluted to ascertain values up to 100,000 pg/ml. A further dilution was not always possible due to the small residual specimen volume. Due to the broad distribution with values below or above our measurement range and for better comparability of IFABP and TFF3, a division was carried out, and values were converted into an ordinal scale by classifying them into categories (Table 1). The categories built were based on the span of the latest pediatric publications (23, 24). In a metaanalysis carried out by Cheng et al. for IFABP as an serological marker in diagnosis for NEC in preterm infants. IFABP values >7,700 pg/ml were the highest cutoff-value for NEC among the included studies. A similar process was used to categorize TTF3 values. Due to the lack of publication on TFF3 in patients with CHD the categories were defined mathematically. The group ranges were defined according to the values and their occurrence in the examined population (Table 1).

**Table 1:** Grouping of the analyzed biomarkers.

Group	IFABP urine	IFABP serum	TFF3 urine	TFF3 serum
1	<40 pg/ml	<40 pg/ml	<31,400 pg/ml	<4,072 pg/ml
2	40–340 pg/ml	40–340 pg/ml	31,400–62,800 pg/ml	4,072–8,144 pg/ml
3	340–750 pg/ml	340–750 pg/ml	62,800–94,200 pg/ml	8,144–12,216 pg/ml
4	750–3,050 pg/ml	750–3,050 pg/ml	94,200–1,25,600 pg/ml	12,216–16,288 pg/ml
5	3,050–5,350 pg/ml	3,050–5,350 pg/ml	1,25,600–1,57,000 pg/ml	16,288–20,360 pg/ml
6	5,350–7,650 pg/ml	5,350–7,650 pg/ml	1,57,000–3,20,000 pg/ml	20,360–36,250 pg/ml
7	>7,650 pg/ml	>7,650 pg/ml	>3,20,000 pg/ml	>36,250 pg/ml

### 2.6 TI-score

Serum **T**FF3 and urinary **I**FABP categories showed the highest correlations considering long-term outcome parameters. In regression analyses only urinary IFABP categories and TFF3 serum categories were significant. Therefore a combined score was created by adding the class ranks of IFABP in urine and TFF3 in serum. Generating a score from 2 (lowest) to 14 (highest class of both markers) by adding the respective category of serum TFF3 and urinary IFABP enables an even more powerful prediction of the outcome parameters.

### 2.7 Statistical analysis

All statistical analyses were performed by SPSS 25.0 and 28.0 (IBM Corp., Armonk, New York, N.Y., USA). Graphics were produced by SPSS 25.0/28.0 and GraphPad Prism 9.0 (Graphpad Software, San Diego, USA). Normal distribution was assessed by using the Kolmogorov–Smirnov test and the Shapiro–Wilk test. Correlations between variables were calculated using Pearson's correlation for metric-scaled variables and Spearman's correlation for ordinally scaled variables.

Paired *t*-test and Wilcoxon rank-sum test were used for within group comparisons. Unpaired *t*-test and Mann–Whitney *U* test were used for between group comparisons.

To compare TTF3 levels and TI-scores depending on the respective STAT-category the Kruskal–Wallis-Test was used. Due to multiple comparisons a *post-hoc* Bonferroni-correction was performed for adjusted *p*-values.

Independent variables significantly associated with time until full enteral nutrition was achieved were identified using univariate logistic regression. Variables with *p* < 0.05 were used for multivariate linear regression modeling. Significant models (*p* < 0.001) were compared for model accuracy using R² and in case of different numbers in variables adjusted R².

Univariate logistic regression analysis was used to identify significant clinical factors (*p* < 0.05) for prolonged enteral feeding among tested variables. The identified covariates were entered into a multivariate logistic regression analysis. Backwards elimination was used to assess which were independently associated with prolonged enteral feeding. The variables used for univariate logistic and linear regression were preselected. For a full list of the tested variables see Tables 3, 4.

A ROC analysis was performed to assess the most accurate cut-off value of the TI-score for predicting prolonged enteral feeding. Overall accuracy of TI score 12 h after surgery for predicting prolonged enteral feeding was represented by area under the curve (AUC). *P*-values less than 0.05 were considered to be statistically significant.

## 3 Results

A total of 85 out of 130 enrolled patients were analyzed. 10 Parents withdrew their permission during the study before sample analysis. 35 patients were excluded before analysis because of incomplete data sets. The remaining 85 patients combined for 99 surgical procedures. Patient characteristics are available in Table 2.

**Table 2:** Patients characteristics.

*N*	85 patients (99 operative episodes)
Sex (male/female)	57 (67%)/28 (33%)
Age at surgery (range)	1 day to 6 years (median 108 days)
Weight at surgery	1.7–40 kg (median 4.835 kg)
CHD complex/simple (25)	79 (79.8%)/20 (20.2%)
STAT Category 1/2/3/4/5	23 (23,2%)/28 (28,3%)/6 (6%)/33 (33,3%)/9 (9%)
Selective cerebral perfusion during surgery yes/no	16 (16,1%)/83 (83,8%)
Surgeries on/off pump	99 79 (79,8%)/20 (20,2%)
Extracardiac shunt yes/no	58 (58.58%)/41 (41.41%)
Shunt dependent body/lung perfusion	50 (overall) 32 (64%)/18 (36%)

### 3.1 Serum and urinary IFABP

73% of preoperative IFABP serum values exceeded the previously reported cut-off value of 336 pg/ml (23). Most values belonged to groups 3–4, with only 2% having values above 7,650 pg/ml (group 7). After the introduction of anaesthesia (T1) IFABP levels increased significantly (*p* < 0.001)with 10% of IFABP values above 7,650 pg/ml. Less than 10% of the included children had normal values after anaesthesia induction. IFABP levels peaked at T2 (after admission to PCICU) with 17% of IFABP values above 7,650 pg/ml and only 5,5% in groups 1 & 2. In the following days, IFABP levels decreased gradually reaching a similar distribution to T0 within 60 h after surgery (23% vs. 27%). Urinary IFABP levels did not increase to the same extent at T1 (56% < 340 pg/ml). During the rest of the study period IFABP categories for urine showed similar course-like serum values, peaking at T2 right after surgery (36% > 7,650 pg/ml) and returning to baseline levels within 60 h after surgery (61% < 340 pg/ml).

### 3.2 Serum and urinary TFF3

Unlike IFABP, presurgery urine values for TFF3 (mean 170.386 pg/ml) decreased after the introduction of anaesthesia (mean 133.384 pg/ml). While rising immediately after surgery (T2) (mean 149.843 pg/ml), urinary values showed another significant decrease (*p* < 0.001) by over 50% until 36 h post-surgery (mean 80.298 pg/ml). At T5, TFF3 levels began to increase again (mean 89.678 pg/ml). TFF3 in serum also dropped from T0 (mean 17.464 pg/ml) to T1 (mean 14.289 pg/ml), while increasing continuously afterwards until 60 h after surgery (mean 16.089 pg/ml).

The ordinal scales of IFABP levels correlated significantly in serum and urine at every time after surgery (T2: rho 0.546 *p* < 0.001; T3: rho 0.486 *p* < 0.001; T4 rho 0.278 *p* = 0.016; T5 rho 0.281 *p* = 0.033). TFF3 categories in serum and urine showed significant correlations (*p* < 0.001) during the whole study period (rho 0,339 −0,645).

### 3.3 Outcome parameters

Urinary IFABP values showed significant correlations at T3 for the duration of invasive ventilation (rho: 0.386), duration of non-invasive ventilation (rho: 0.447), length of stay (rho: 0.443) and time until complete enteral nutrition (rho: 0.523), all at a significance level of *p* < 0.001.

For TFF3, serum category correlations were stronger for the same outcome parameters than urinary categories across all examined time points. Like IFABP, correlations with duration of invasive ventilation (rho: 0.440), duration of non-invasive-ventilation (rho: 0.410), length of stay (rho: 0.475) and time until full enteral nutrition (rho: 0.465) were the strongest at T3 (*p* < 0.01).

Urinary IFABP and serum TFF3 also correlated during the whole study period. (T0: 0.645; T1: 0.339; T2: 0.408; T3: 0.498; T4: 0.481; T5: 0.435; *p* < 0.01).

### 3.4 TI-score

The TI-score correlated significantly with the examined long-term outcome parameters from (T1)–(T5). Like the single biomarkers, the newly generated score showed the highest correlations 12 h after surgery (T3). (Spearman's rho 0.497 for the duration of invasive ventilation; 0.485 for duration of non-invasive ventilation; 0.61 for time until full enteral nutrition and 0.532 for length of hospital stay with *p* < 0.01). The TI-score showed stronger correlations with long-term outcome parameters than the biomarkers on their own.

### 3.5 Clinical parameters

IFABP in urine and TFF3 in serum showed significant positive correlations with VIS-score and serum lactate. Urinary IFABP at T3 correlated with VIS from 6 h–72 h after surgery (*p* < 0.001–0.0015). Serum TFF3 correlated at T2 with VIS-scores from 24 h–72 h after surgery (*p* = 0.008–0.048). Using the TI-score at T2 improved the correlations (*p* < 0.001–0.003). The TI-score correlated significantly with serum lactate levels (from 6 h–72 h after surgery) from T1–T5. T1 (rho 0.349; *p* = 0.005). T2 (rho 0.384; *p* < 0.001). From T3–T5 (rho 0.419/0.499/0.564; *p* < 0.001).

### 3.6 Clinical variables associated with TI score 12 h after surgery

We conducted univariate regression analysis to identify (*p* < 0.05) significantly associated with TI score 12 h after surgery (see Table 3). The significant variables were entered in multivariat linear regression modeling. Forward and backward procedures were conducted do identify those independently associated with higher TI-scores 12 h at T3. The only covariates independently associated with TI score 12 h after surgery were minimal temperature during surgery (*p* 0.002), STAT category (*p* 0.002) and ductal dependent circulation (*p* 0.017).

**Table 3:** Univariate regression for TI-score 12 h after surgery. Bold values represent statistically significant values (*p* < 0.05).

Independent variable	*p*-value
STAT Category	**<0** **.** **001**
Ductal dependent circulation	**<0** **.** **001**
Age at surgery	**<0** **.** **001**
Time of Intubation	**0** **.** **008**
Time of non-invasive ventilation	0.41
Surgery with CPB	0.87
CPB time	**<0** **.** **001**
Aortic-cross-clamp time	0.325
Reperfusion time	**<0** **.** **001**
Minimal temperature during surgery	**<0** **.** **001**
Selective cerebral perfusion	0.48
VIS-score	**0** **.** **037**
Diarrhea	0.56
Gastric residual volume	0.41
Oral intake in first 24 h	0.23

### 3.7 Enteral feeding and gastrointestinal complications

Gastrointestinal complications and signs of feeding intolerance (emesis, gastric residual volume, diarrhea) showed no association with the tested biomarkers. There were no cases of NEC in our study population.

There was no significant difference between different types of nutrition (breast milk, follow-up milk, pre-milk) and their respective biomarker values. In general we do not prescripe TPN post surgery. Infants on partial parenteral nutrition (PPN) received only tea as oral intake and did not differ in biomarker levels from those who received enteral nutrition.

The TI-score after surgery and the amound of fed milk 48–72 h post-op showed significant inverse correlations (T2 rho: −0.425; *p* < 0.001; T3 rho: −0.471; *p* < 0.001; T4 rho: −0.478 *p* < 0.001; T5: rho: −0.303; *p*: 0.026).

We performed a multivariable linear regression analysis to identify those factors associated with time until full enteral nutrition. Variables significant in univariate analysis were added to the multivariate modeling. For a full list of the tested variables in univariate analysis see Table 3. The TI-score 12 h after surgery was independently associated with delayed enteral feeding progress. Due to high collinearity between the different VIS-scores we chose the VIS-score with the highest significance (VIS 24–48 h) for final modeling. The most accurate model (adjusted *r*^2^ = 0.764) included invasive (*p* < 0.001) and non-invasive ventilation duration (*p* < 0.001), VIS-score 24–48 h (*p* < 0.001) and TI-score 12 h after surgery (*p* 0.036).

**Figure 1:**
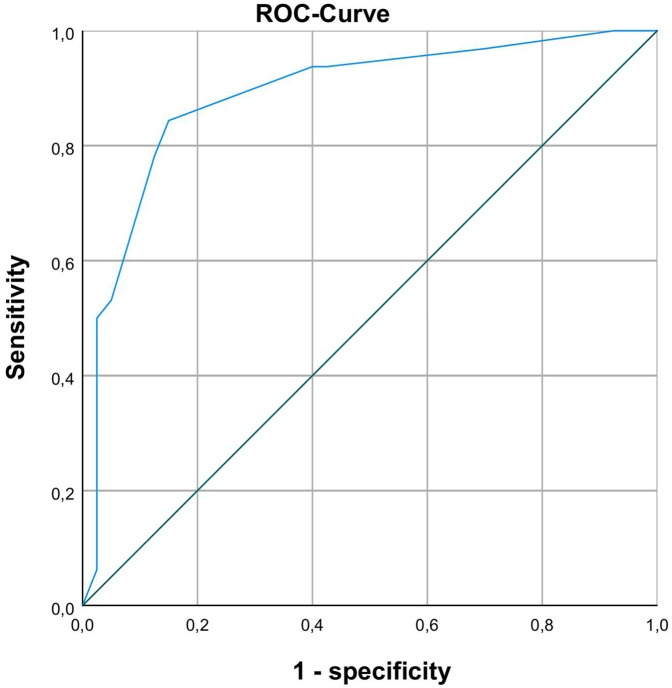
ROC-analysis with AUC 0.888 for differentiating between normal and prolonged enteral feeding process with TI-scores 12 h after surgery.

Binary logistic regression analysis was performed to identify clinical markers associated with prolonged enteral feeding. 6 days until complete enteral nutrition (100 mg/kg bodyweight/day) was defined as a cut-off point for prolonged enteral feeding. Independent variables associated with prolonged enteral feeding were identified by using univariable logistic regression and entered into the multivariate models. For a full list of tested variables in univariate analysis see Table 4. In a multivariable logistic regression model only duration of invasive ventilation (*p* = 0.028) and TI-score 12 h after surgery (*p* = 0.009) proved to be significant independent predictors (for further details see Tables 5, 6) for prolonged enteral feeding (*n* = 72 AUC 0.888; Figure 1). A reasonable cut-off value for TI-score 12 h after surgery seems to be 6.5 with a sensitivity of 84.4% and a specificity of 85%.

**Table 4:** Univariate linear regression for time until full enteral nutrition (100 ml/kg) was achieved after cardiac repair surgery. Bold values represent statistically significant values (*p* < 0.05).

Independent variable	*p*-value
STAT Category	**<0** **.** **001**
Age at surgery	**0** **.** **026**
Time of Intubation	**<0** **.** **001**
Time of non-invasive ventilation	**<0** **.** **001**
Surgery with CPB	0.72
Aortic-cross-clamp time	0.22
Minimal temperature during surgery	**0** **.** **003**
Selective cerebral perfusion	0.28
VIS-score 24–48 h after surgery	**<0** **.** **001**
TI-score 12 h after surgery	**0** **.** **004**
Vomiting	0.19
Diarrhea	049
Gastric residual volume	0.63

**Table 5:** Univariate logistic regression for predicting prolonged enteral feeding (>6 days). Bold values represent statistically significant valueso (*p* < 0.05).

Independent variable	OR (95% CI)	*p*-value
STAT Category	2.227 (1.555–3.191)	**<0** **.** **001**
Age at surgery	0.993 (0.988–0.997)	**0** **.** **002**
Time of Intubation	1.030 (1.017–1.044)	**<0** **.** **001**
Time of non-invasive ventilation	1.002 (1.000–1.005)	0.084
Surgery with CPB	1.200 (0.453–3.180)	0.714
Aortic-cross-clamp time	1.013 (1.002–1.023)	**0** **.** **016**
Minimal temperature during surgery	0.726 (0.600–0.878)	**<0** **.** **001**
Selective cerebral perfusion	10.156 (2.148–48.015)	**0** **.** **003**
VIS-score 24–48 h after surgery	1.125 (1.054–1.201)	**<0** **.** **001**
TI-score 12 h after surgery	1.894 (1.422–2.521)	**<0** **.** **001**
Vomiting	0.191 (0.028–1.313)	0.092
Defecation frequency	0.638 (0.419–0.969)	**0** **.** **035**
Gastric residual volume 24–48 h after surgery	1.039 (1.003–1.077)	**0** **.** **033**

OR, odds ratio; CI, confidence interval; VIS-score, vasoactive-inotropic score; TI-score, TFF3 IFABP.

**Table 6:** Multivariate model for predicting prolonged enteral feeding (>6 days). Bold values represent statistically significant values (*p* < 0.05).

Independent variable	OR (95% CI)	*P* Value
Time of Intubation	1.017 (1.002–1.032)	**0** **.** **028**
TI-score 12 h after surgery	1.613 (1.128–2.305)	**0** **.** **009**

### 3.8 Hemodynamics

**Figure 2:**
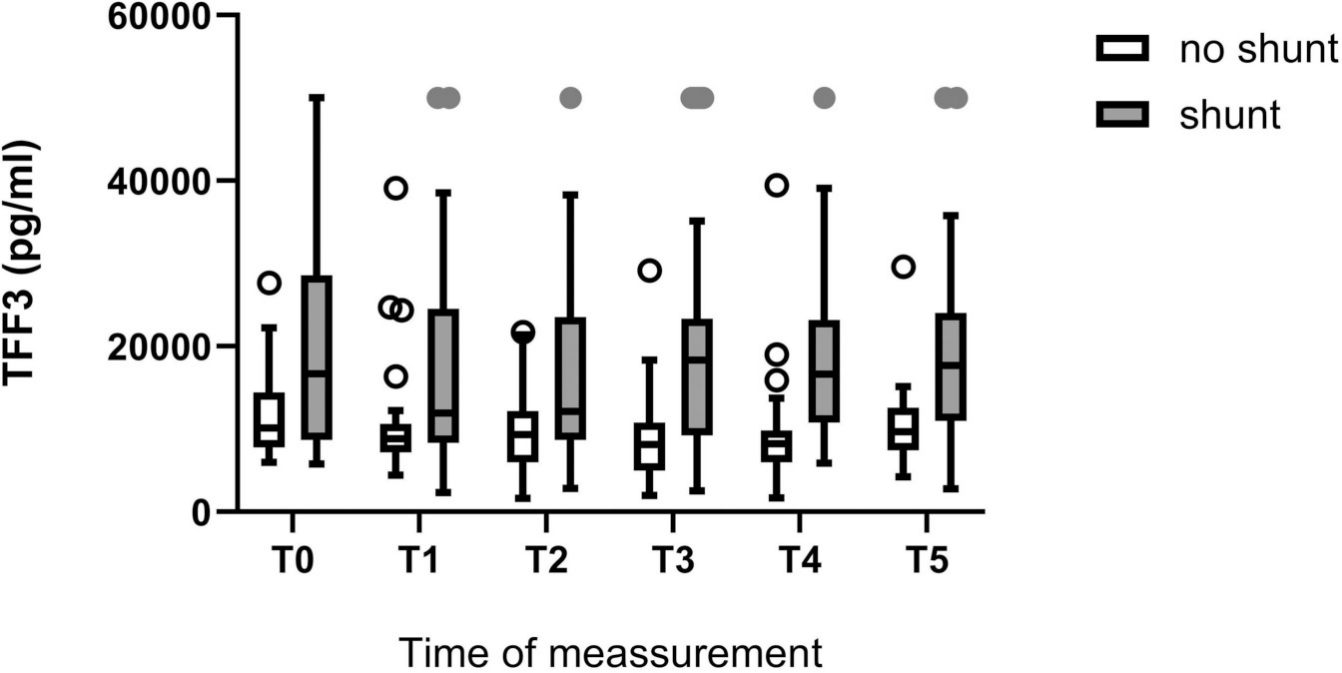
Comparison of serum TFF3 levels in CHD children with and without extracardiac shunt. T0: *p* = 0.045 T1: *p* = 0.05 T2: *p* = 0.025 T3: *p* = 0.01 T4: *p* = 0.01 T5: *p* = 0.042.

The two biomarkers significantly differ in children with and without extracardiac vascular shunts. Those with extracardiac shunts showed significantly higher serum TFF3 levels (see Figure 2). The TI-score was also able to discriminate between these two conditions and was also superior to the individual measurements here *p* < 0.02 at all times). There was no statistical significant difference between ductal dependent systmic perfusion and ductal dependent pulmonary circulation in biomarker levels or TI-score.

### 3.9 Simple vs. complex congenital heart defects

**Figure 3:**
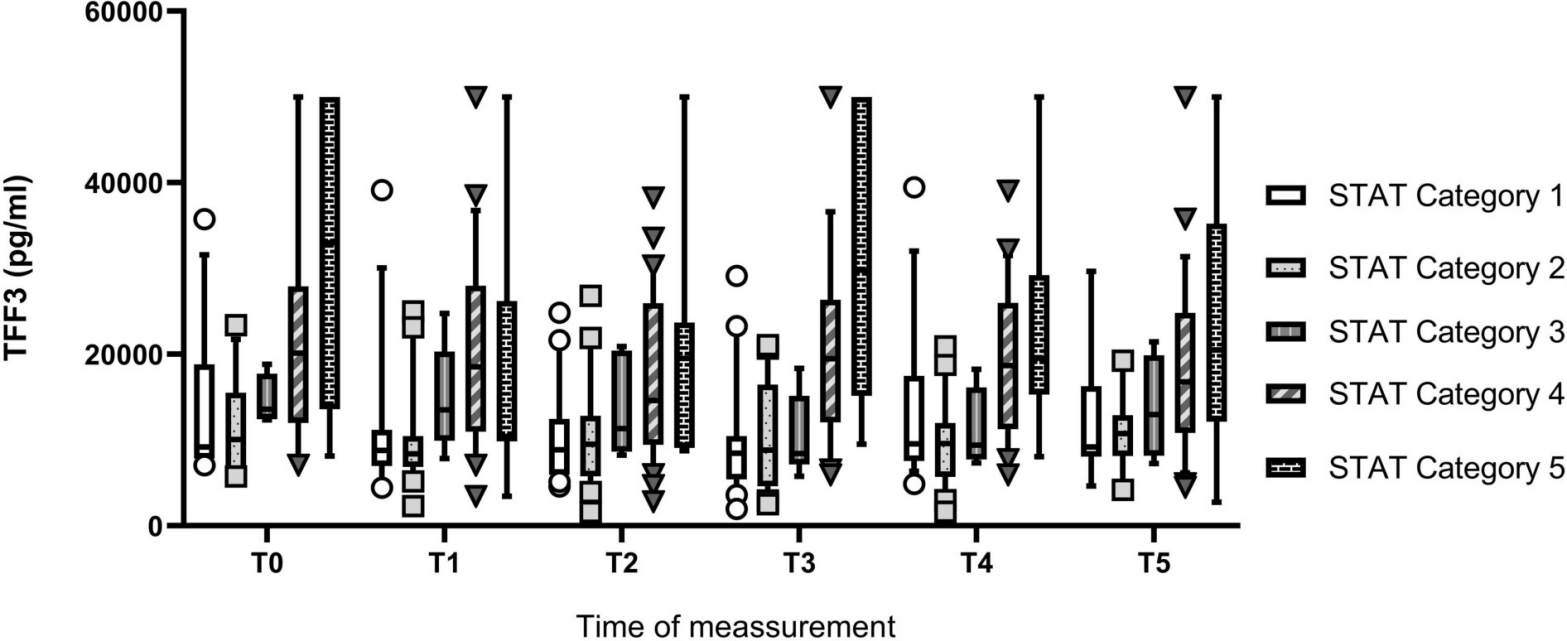
Comparison of serum TFF3 levels in CHD children depending on their STAT category. Significant differences after Bonferroni correction: T0: 2&5 (*p* 0.048). T1: 1&4 (*p* 0.02); 2&4 (*p* 0.014). T2: 1&4 (*p* 0.044) 1&5 (*p* 0.026); 2&4 (*p* 0.029); 2&5 (0.024). T3: 1&4 (*p* 0.001); 1&5 (*p* 0.003); 2&4 (*p* 0.011); 2&5 (*p* 0.011). T4: 1&4 (*p* 0.019); 1&5 (*p* 0.015); 2&4 (*p* 0.011); 2&5 (*p* 0.015). T5: 2&4 (*p* 0.01); 2&5 (*p* 0.022).

Children with complex (25) cardiac defects showed significantly higher IFAPB categories in urine (*p* = 0.045) with a wide range from 1–7 before surgery than those with simple defects (all values below 340 pg/ml). For TFF3, categories also were significantly higher in children with complex CHD from T2–T4 (p: T2: 0.022; T3: 0.001; T4: 0.021). They also had significant higher TI-scores from T1–T5 (p: T1: 0.02; T2: 0.036; T3: <0.001; T4: 0.018; T5: 0.046). The different STAT categories showed significant differences in their mean ranks for serum TT3 levels from T0–T5 (see Figure 3) and for TI-score from T1–T5. After Bonferroni correction for multiple comparisons the only differences that remained significant for serum TFF3 were between STAT category 1&4; 1&5, 2&4 and 2&5. For TI-score significant differences were found between STAT categories 1&4 (T1–T4); 1&5 (T1–T5); 2&4 (T1, T3–5) and 2&5 (T2–T5). A sub-analysis to decrease the heterogeneity of our study group further by grouping patients with the same typ of CHD showed no significant differences between a specific heart defect and the tested biomarkers or TI-score (data not shown).

## 4 Discussion

This study evaluated the combination of IFABP and TFF3 in urine and blood as perioperative biomarkers in children with congenital heart disease (CHD). Rather than attempting to provide definitive diagnostic performance, our analysis focuses on describing the perioperative kinetics of these biomarkers and exploring their association with clinical outcomes.

Our findings align with recurrent themes across the CHD literature: (i) preoperative IFABP elevation consistent with impaired splanchnic perfusion, (ii) sharp postoperative rises after cardiopulmonary bypass (CPB), and (iii) associations with adverse gastrointestinal outcomes. These patterns have been reported by Typpo et al. (11) and Watson et al. (12) and are reinforced by recent studies linking postoperative IFABP to feeding intolerance and NEC (Owens et al., 2024; Ali et al., 2023). Building on this foundation, our dual-matrix (serum/urine) and dual-pathway (injury/repair) approach extends prior work by examining IFABP together with TFF3.

We explicitly interpret circulating IFABP as a surrogate of enterocyte injury rather than a direct histologic measure. This interpretation is supported by human intestinal ischemia models (14, 26) as well as porcine (27, 28) and mouse (29) models. Heida et al. (15) showed a strong association in their preterm NEC-cohort between IFABP levels in serum/urine and the length of intestinal resection. Ali et al. (30) further demonstrated increased IFABP at the time of clinical NEC in neonates after CHD surgery, and Owens et al. (31) reported higher postoperative IFABP together with tight-junction proteins (claudin-2, claudin-3) in children who developed feeding intolerance, strengthening the biological plausibility of IFABP as an epithelial-injury marker. However it must be noted that injury in that context lacks a clear definition. Is injury defined by presentation of gastrointestinal symptoms/signs of feeding intolerance, by increases in biomarker levels or by histological changes? Different studies (14, 29, 32) demonstrated that a short (15–30 min) period of ischemia is enough to cause significantly increasing IFABP levels while presenting no relevant macroscopic damage. On a histological level subepithelial spaces appeared while the epithelial lining was still unimpaired. This has to be considered when using IFBAP as a surrogate for intestinal injury.

Serum and urinary IFABP demonstrated divergent kinetics. Serum concentrations rose rapidly, capturing acute peri-induction and intraoperative events, whereas urinary IFABP changed more slowly, likely reflecting renal handling and cumulative injury. Unfortunately due to classification into groups we were not able to normalize urinary biomarker levels to creatinine. Changes in diuresis during our study period can therefore confound our result. This has to be taken into account when comparing our results to other studies.

Interestingly prior studies (11, 31) sampled pre-operative after anesthesia induction. We observed that induction coincides with early IFABP increases. On the one hand this could explain even higher pre-operative values in their study cohort. On the other hand it indicates that anesthetic-related reductions in systemic vascular resistance may transiently impair splanchnic perfusion and promote early enterocyte injury. This finding opens a potential therapeutic window: optimizing hemodynamics and perfusion during anesthetic induction may mitigate early intestinal epithelial compromise before surgical intervention begins.

Serum TFF3 was markedly elevated preoperatively and rose until 60 h postoperatively, consistent with restitution biology (17, 33). However, we also acknowledge an alternative explanation: reduced renal clearance may contribute to higher circulating TFF3 particularly in shunt-dependent lesions with lower baseline GFR. Owens (31) reported reduced glomerular function and persistent barrier dysfunction after CPB, which may reinforce this interpretation. The postoperative decline in urinary TFF3 is compatible with clearance effects. Importantly, Ali (30) demonstrated increased circulating TFF3 (together with TFF2 and IFABP) at clinical NEC onset in neonates with CHD, supporting its association with true intestinal injury. Taken together, serum TFF3 likely reflects both reparative activity and altered clearance, which must be considered in interpretation.

### 4.1 Combined biomarkers as a potential predictor for postoperative course

Both IFABP and TFF3 showed significant correlations with clinical parameters such as duration of intubation, time on non-invasive ventilation, hospital length of stay, and time to full enteral nutrition. Notably, urinary IFABP had better associations when categorized into diagnostic ranges, while TFF3 demonstrated more consistent predictive value in serum. These findings align with their respective pharmacokinetics and release mechanisms: IFABP reflects acute epithelial disruption, whereas TFF3 reflects a more prolonged reparative response. Combining both markers into a composite TI score improved correlations with outcomes. The strongest correlations were observed at T3 (12 h post-surgery), suggesting that this timepoint captures both the extent of intestinal injury and the initial reparative response and may serve as a critical window for prognostication.

### 4.2 Nutrition

The literature on feeding outcomes is heterogeneous. Typpo (11) reported lower IFABP with higher intolerance scores, but also found early enteral feeding linked to improved tolerance. Watson (12) did not see an association with time to feeds but did report a link between postoperative IFABP and later NEC. In a more homogeneous neonatal/young infant cohort, they did not find an association with time to enteral feeding (which tracked clinical factors such as cardiovascular support), but did show an independent association between postoperative IFABP and later clinical NEC. Owens (31) linked higher postoperative IFABP and tight-junction proteins (claudin-2/-3) to feeding intolerance, while Ali (30) observed IFABP elevations at clinical NEC onset in postoperative CHD patients. In our study, the TI-score provided stronger prognostic associations with feeding outcomes, suggesting complementary contributions of injury (IFABP) and repair/clearance-affected (TFF3) biology. The diagnostic performance of the TI-score at 12 h post-surgery (AUC = 0.888) surpassed that of other biomarkers for enteral feeding like serum amyloid A in NEC patients (34), possibly due to higher specificity of IFABP and TFF3 for intestinal injury. Enteral feeding in children post-cardiac surgery is currently guided by clinical judgement, with significant inter-institutional variability (35, 36). Current data suggest that biomarkers can play a role in predicting progress in enteral feeding but further studies are necessary to find the most suitable biomarker or a combination thereof.

The TI-score could provide an objective biomarker-based tool to identify children at risk for delayed enteral feeding and help tailor nutrition protocols. A single measurement at 12 h post-surgery may suffice, minimizing sample burden while maximizing prognostic value.

### 4.3 Vasoactive inotropic score

The treatment with different vasoactive medications during surgery and at PICU affects intestinal perfusion and therefore influences IFABP and TFF3. We also demonstrated significant correlations between IFABP/TFF3 and VIS-score, although the VIS-score was not independently associated with higher TI-scores. This contrasts with studies by Typpo (11) and Watson (12), likely due to our institution's restrictive use of vasopressin and epinephrine. Both agents are known to impair splanchnic perfusion more severely than other catecholamines (37–42). This suggests that institutional vasoactive practices may influence biomarker expression and thus need to be accounted for in future multicenter studies.

### 4.4 Differences in cardiac defects

Subgroup analysis revealed that both TFF3 and TI-score distinguished between children with and without shunt-dependent lung or body perfusion. Children with complex CHD requiring shunts showed significantly higher biomarker levels preoperatively, with broader interindividual variability. This underscores the physiological heterogeneity in this patient population and suggests that biomarkers like TFF3 may aid in stratifying risk even before surgery. However the above mentioned lower GFR in shunt-depentent lesions may elevate circulating TFF3 due to reduced clearance, limiting subgroup (shunt vs. no shunt) interpretation. We were not able to show differences between ductal dependent systemic and pulmonary circulation. Nevertheless this aspect should be further analysed in studies with larger cohorts. Patients with ductal dependent systemic circulation might be at a higher risk for gastrointestinal complication. The lack of significant differences in our cohort could also be confounded by the small number of patients. TFF3 and TI-Score were also able to discriminate between low risk (STAT 1&2) and high risk (STAT 4&5) surgical procedures. The small subgroups of STAT categories haven to be taken into account here, when comparing these finding with our studies. It must be noted that TFF3 is not specific to intestinal epithelium. It is also expressed in the respiratory tract (43), and higher levels in shunt-dependent CHD may partly reflect pulmonary epithelial injury due to volume overload. Current ELISA assays do not differentiate the source of TFF3, highlighting the need for more specific assays in future studies.

## 5 Conclusion

IFABP and TFF3 are promising, mechanistically grounded biomarkers that reflect intestinal injury and repair following cardiac surgery in children with CHD. The TI-score, particularly at 12 h post-surgery, offers valuable prognostic information for clinical outcomes, including duration of mechanical ventilation and time to full enteral nutrition. Future studies should explore the integration of these biomarkers into perioperative care protocols and assess their utility in guiding nutritional and hemodynamic management in this high-risk population.

## 6 Limitations

Our study has several limitations. As a single-centre trial, generalizability is restricted. The cohort heterogeneity in terms of cardiac defects resulted in small subgroup sizes, limiting statistical power. We evaluated biomarker levels as surrogate for intestinal injury, while not assessing for actual histological changes or intestinal epithelial damage. Even though age was not independently associated with progress in enteral nutrition or biomarker levels, the wide range of age in our study cohort still makes it a potential confounder of our results. The age of our data-set (2015–2017) limits the transferability especially as fast-track procedures have increased until today. The absence of a standardized feeding protocol in the ICU introduces variability in outcome assessment. We were not able to account for all clinical factors influencing the decision making process in enteral feeding (such as the general clinical impression of the patient as well as personal work habits and experience of the physicians and nursing staff) hence these could confound our results. The fact that we were not able not correct the urinary biomarker levels for urine creatinine concentration limits the validity of the urinary biomarker analysis. Especially as impaired renal clearance can result in increased circulating biomarkers like TFF3. Finally, limited blood volume in pediatric patients constrained the availability of samples for repeated measurements and dilutions.

## Data Availability

The raw data supporting the conclusions of this article will be made available by the authors, without undue reservation.
